# The Effect of Vanadium Content Coupling with Heat Treatment Process on the Properties of Low-Vanadium Wear-Resistant Alloy

**DOI:** 10.3390/ma15010285

**Published:** 2021-12-31

**Authors:** Tao Jiang, Shizhong Wei, Liujie Xu, Cheng Zhang, Xiaodong Wang, Mei Xiong, Feng Mao, Chong Chen

**Affiliations:** 1 School of Materials Science and Engineering, Henan University of Science and Technology, Luoyang 471003, China; tedivy@163.com; 2 National Joint Engineering Research Center for Abrasion Control and Molding of Metal Materials, Henan University of Science and Technology, Luoyang 471003, China; wmxlj@126.com (L.X.); zhangch06@126.com (C.Z.); 18567617098@163.com (X.W.); xiongmei_1327@163.com (M.X.); maofeng718@163.com (F.M.)

**Keywords:** abrasive wear, vanadium carbide, heat treatment, high chromium cast iron

## Abstract

The development of wear-resistant materials with excellent properties is of great research value in the manufacturing industry. In this paper, a new kind of low-vanadium wear-resistant alloy was designed and characterized to unveil the influence of vanadium content coupling with heat treatment on the microstructure, hardness, and abrasive wear property. The performances of commercial high chromium cast iron (HCCI) and the new low-vanadium wear-resistant alloy are compared. The alloy with 3 wt.% vanadium quenched at 900 °C and tempered at 250 °C, possessing VC, Mo_2_C, and M_7_C_3_ distributed in the martensite matrix, displayed a wear resistance two times better than the HCCI. The results showed that the increase of vanadium content from 0.98 wt.% to 3.00 wt.% resulted in a decrease in the size of M_7_C_3_ and a more homogeneous distribution of M_7_C_3_. VC with a bimodal distribution is effective for impeding grooving or indenting by abrasives because of their high hardness, which plays a vital role in improving the wear resistance of low-vanadium wear-resistant alloy.

## 1. Introduction

Wear-resistant materials are important consumables in the manufacturing industry and are widely used in mining, metallurgy, and cement industries [1,2,3]. An assortment of these parts includes: construction and mining machinery parts (excavators’ teeth and teeth covers) and parts of grinders and mills for stone, ore, coal, and minerals (balls, hammers, impact plates, mill linings, and separation grids, etc.). Due to the direct contact with materials to be processed, wear-resistant materials bear huge crushing force and friction [4]. Service life is directly related to the work efficiency and production cost of construction machinery [5]. Therefore, it is particularly important to extend the service life of wear-resistant alloy parts. The performance of wear-resistant materials needs to be improved to meet the service life requirements of large-scale equipment. Otherwise it will result in large consumption of wear-resistant materials, low equipment operation efficiency, and high production costs, which severely restrict the development of equipment manufacturing in the direction of large-scale, high-efficiency, and continuity. Improving the service life of wear-resistant materials has become one of the important tasks for improving the overall competitiveness of the manufacturing industry [6,7,8,9].

Factors including physical design, material selection, and assembly process should be taken into consideration to improve the service life of wear-resistant alloys. Among these factors, proper material selection is one of the most effective ways to prolong the service life of wear-resistant alloys. Conflicting characteristics of high hardness for maximum wear resistance and adequate ductility to avoid catastrophic brittle failure in application must be balanced with reference to cost. A range of materials has been developed for this purpose, which include abrasion resistant steels, non-metallics, and alloyed white cast irons [3]. Of these materials, conventional wear resistant materials for impact wear applications are Hadfield-type Mn-steels and high chromium white cast irons. Due to its excellent toughness and significant work hardening characteristics, Hadfield-type Mn-steel has excellent wear resistance under high stress and high-impact wear conditions and is widely used [10,11]. However, Hadfield-type Mn-steel cannot give full play to its advantages under low impact and low stress wear conditions. Chromium white cast iron has high initial hardness and excellent wear resistance under low impact and low stress conditions. As a kind of chromium white cast irons, high chromium cast iron (HCCI) is an anti-wear material with excellent performance [12,13]. It has much higher wear resistance than alloy steel and much higher toughness and strength than ordinary white cast iron. At the same time, it also has good high temperature resistance and corrosion resistance [14], combined with convenient production and moderate cost, and it is known as one of the best anti-abrasive materials currently. These materials have been explored and used for decades in various applications. However, the toughness of HCCI is poor, and the wear resistance needs to be further improved. Therefore, new high-performance wear-resistant alloys can still be expected.

As a new generation of wear-resistant materials, high vanadium high-speed steel is a new steel grade formed by reducing the content of tungsten, increasing the content of vanadium, and adding alloy elements such as molybdenum, chromium, and niobium on the basis of traditional high-speed steel [1,15]. Compared with high chromium cast iron, the abrasive wear performance, impact wear performance, and rolling wear performance of high vanadium high-speed steel have significant advantages [16,17,18,19,20,21]. The current research on high vanadium high-speed steel mainly involves the study of alloy composition [22,23], heat treatment process [18,24], and wear performance and mechanisms [16,21,25]. Due to the high vanadium content in high vanadium high-speed steel (usually 3.0–10.0 wt.%), the material price is very expensive, which is not conducive to the wide application of high-vanadium high-speed steel.

Because of its cost-effectiveness, the research and application of low-vanadium wear-resistant alloys are much anticipated. For this purpose, a new low-vanadium wear-resistant alloy system was designed in this paper. The microstructure characteristics and performance of the new low-vanadium wear-resistant alloy under various vanadium contents coupling with heat treatment process conditions were studied. The relationship between composition, process, structure, and performance was analyzed and explained. In addition, a low-vanadium wear-resistant alloy with outstanding cost performance was developed in this research, which provides an alternative material for the field of wear-resistant materials.

## 2. Materials and Methods

Low-vanadium wear-resistant alloys were prepared by ordinary sand casting in a medium frequency induction furnace, and then the ingots were annealed at 550 °C for 2 h. The commercial high chromium cast iron (HCCI) is a commercial alloy taken as the contrastive object here. The measured composition of the three kinds of wear-resistant alloys with different vanadium content and the composition of HCCI are shown in Table 1.

To study the effect of heat treatment process parameters on the performances of low-vanadium wear-resistant alloys, heat treatments with different quenching temperatures and tempering temperatures were carried out. Austenitizing temperatures of 850 °C, 900 °C, 950 °C, 1000 °C, and 1050 °C were selected, with the holding time of 30 min. Due to the high carbon content of the alloys, oil quenching is selected for all samples. Tempering temperatures of 150 °C, 250 °C, 350 °C, 450 °C, and 550 °C were selected, with the holding time of 2 h followed by air cooling.

To reveal the microstructure, metallographic samples were cut, ground, and polished following standard procedures. Four percent nitric acid alcohol solution was used to reveal the microstructures. Secondary electron (SE) images of the microstructure and worn surface were observed by field emission scanning electron microscope (JSM-IT800) through a secondary electron detector. Backscattered-electron (BSE) images obtained by a backscattered electron detector in JSM-IT800 was used to distinguish between different phases. Energy dispersive spectroscopy (EDS) analysis was carried out for elemental and chemical analysis by Oxford Ultim^®^ Max 40. The macrohardness of the specimen was tested by a Rockwell hardness tester (HR-150A) with the load of 150 kg. The microhardness was measured in microscale using an HVS-1000A microhardness tester under an applied load of 0.2 kg. For the convenience of comparison with macrohardness, the test results of microhardness are converted into the corresponding HRC values through the built-in software of the device. Specimens were all polished before hardness tests. Averages of 5 points of testing results is taken as the final hardness value.

The abrasive wear test was carried out on a pin-on-disk (type ML-100) abrasion tester. The working principle of the abrasion tester is described in detail in [26]. The specimens to be tested acted as pins. The diameter of the specimen is 6 mm and the height is 20 mm. The silica sandpaper used for the test is waterproof sandpaper with a particle diameter of 53 μm. The force loaded on the sample during test is 10.0 N. Before test, each specimen was grinded for 3 min to make the surface of the specimen as smooth as possible. Each sample was reciprocated by 15 sets of movement each time, and then wear weight loss was measured. The weight loss is obtained by subtracting the mass of the sample after wear from the mass of the sample before wear. The weight loss of the specimen was measured by TG328B analytical balance with a range of 0~200 g and a relative accuracy of 0.1 mg. The reported weight loss values are the averages of three measurements.

## 3. Results

### 3.1. Microstructure of the Annealed Samples

**Figure 1:**
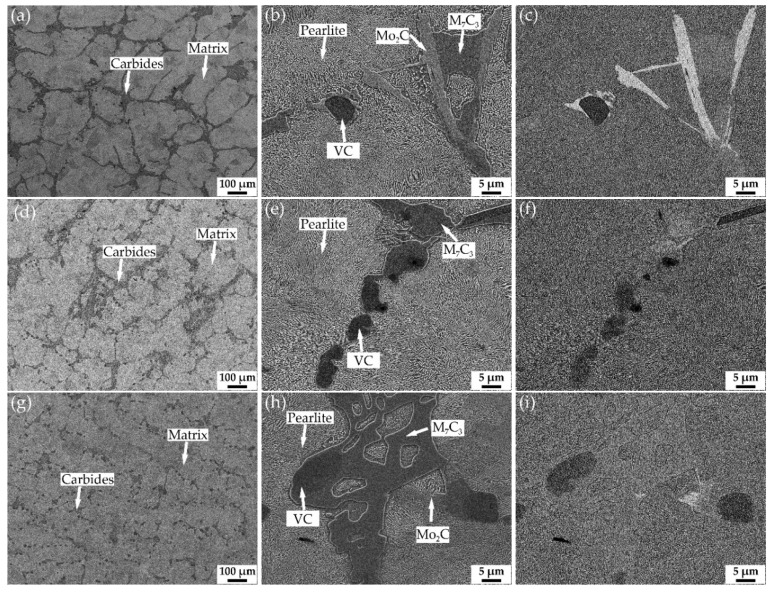
SEM images of the annealed specimens, SE images of V1: (**a**,**b**), BSE image of V1: (**c**), SE images of V2: (**d**,**e**), BSE image of V2: (**f**), SE images of V3: (**g**,**h**), BSE image of V3: (**i**).

Figure 1 shows the SEM images of low-vanadium wear-resistant alloys after annealing. Figure 1a,b,d,e,g,h are secondary electron (SE) images. And Figure 1c,f,i are backscattered electron (BE) images. According to our previous research and the BSE images of V1, V2, and V3, it can be inferred that the types of carbides distributed in the pearlitic matrix are M_7_C_3_, VC, Mo_2_C [17,19]. As shown in Figure 1a,d,g, the volume fraction of eutectic carbide M_7_C_3_ is dominant among all carbides. It can be seen from Figure 1b,e,h that the matrix of the three alloys are all pearlite. The volume fraction of eutectic carbide M_7_C_3_ in V1 and V2 is significantly higher than that of V3, and the size of M_7_C_3_ in V1 and V2 is relatively large, which results in forming carbide network. In comparison, M_7_C_3_ evenly distributed in the matrix of V3 are small. Rod-shaped Mo_2_C and blocky VC are generally attached to other carbides. The mass fraction of VC can be obtained by the mass fraction of V in the alloy because vanadium exists in the alloy in the form of vanadium carbide. The density of VC is about 5.8 g/cm^3^ according to PDF#65-8074. The density of the alloy is about 7.8 g/cm^3^. Based on these data, the volume fraction of VC can be estimated. The volume fractions of VC in V1, V2, and V3 are 1.6%, 3.2%, and 4.8%, respectively.

### 3.2. The Effect of Quenching Temperature on Hardness

**Figure 2:**
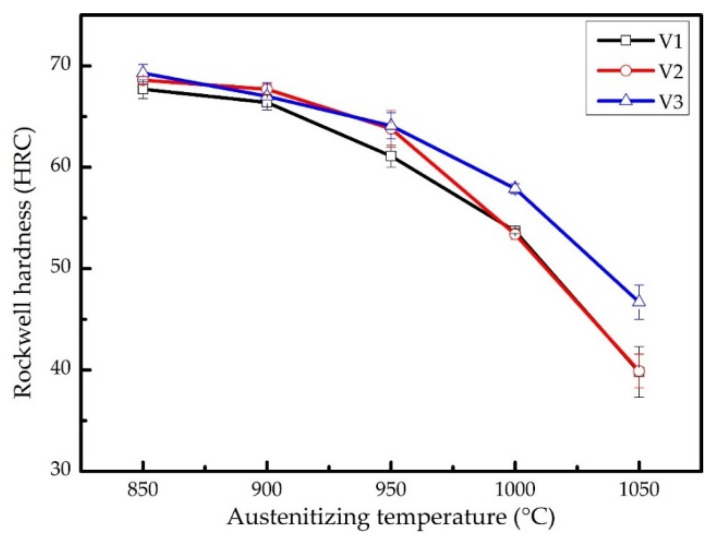
The macro-hardness of the samples after quenching at various temperatures.

Figure 2 shows the macrohardness of the samples after quenching at various temperatures. It can be seen that the macro-hardness of the three samples decreases as the quenching temperature increases. This is because the amount of carbides dissolved into austenite increases as the quenching temperature increases, which leads to the increase of carbon content of the austenite before quenching. The *M*_s_ temperature decreases as the content of the austenite increases, resulting in more retained austenite after quenching. The carbon content in the matrix of V3 is the lowest among the three samples because of a large amount of VC. Thus, the macrohardness of V3 decreases more slowly than that of the other two. Weighing up the hardness and toughness, 900 °C was chosen as quenching temperature for the three alloys in the next abrasive wear test.

### 3.3. Effect of Vanadium Content on Microstructure and Wear Properties

**Figure 3:**
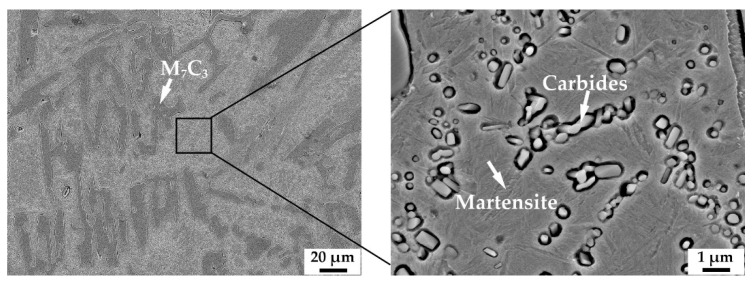
SEM micrograph of HCCI and corresponding magnified micrograph.

**Figure 4:**
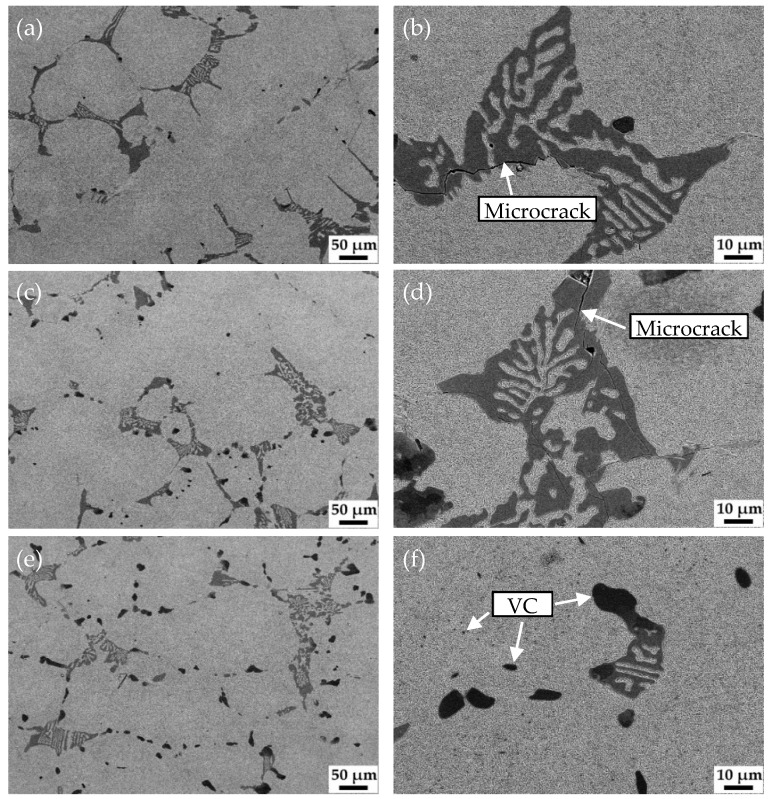
SEM images of the samples with different vanadium contents after quenching at 900 °C and tempering at 250 °C, V1: (**a**,**b**), V2: (**c**,**d**), V3: (**e**,**f**).

Figure 3 shows the micrograph of HCCI and the corresponding magnified micrograph. The microstructure of HCCI is irregular M_7_C_3_ and martensite matrix with fine carbides. Figure 4 shows the microstructure of the samples with different vanadium content after quenching at 900 °C and tempering at 250 °C. It can be seen that M_7_C_3_, Mo_2_C and VC remained unchanged after the austenitization treatment. The matrix microstructure of all samples changed from pearlite to tempered martensite with undissolved carbides. It is worth noting that there were many microcracks formed in some coarse eutectic carbides in V1 (Figure 4b) and V2 (Figure 4d) caused by quenching stress. The carbon content in the matrix of V3 is relatively low, and the size of the eutectic carbide M_7_C_3_ is small, so there is almost no quenching crack (Figure 4f). In addition, a large number of submicron VC are distributed in the matrix of V3, which supposed to induce the high hardness.

**Figure 5:**
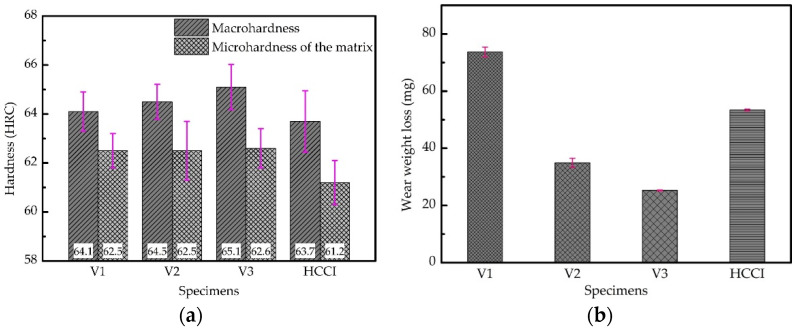
Comparison of macrohardness, microhardness of the matrix, and wear resistance between low-vanadium wear-resistant alloys and HCCI: (**a**) macrohardness and microhardness of the matrix comparison, (**b**) wear resistance comparison.

Figure 5 shows the comparison of macrohardness, microhardness of the matrix, and wear resistance between low-vanadium wear-resistant alloys and HCCI. As can be seen from Figure 5a, the macrohardness of the samples increases slightly as the content of vanadium increases. And the macro-hardness of the three low-vanadium wear-resistant alloys are all higher than HCCI. There is almost no difference in the hardness of the matrix of V1, V2, and V3, as can be seen in Figure 5a. As shown in Figure 5b, the wear resistant property also increases with the increase of vanadium content, and the abrasive wear performance exceeds that of HCCI when the vanadium content exceeds 2 wt.%.

### 3.4. Effect of Tempering Temperature on Microstructure and Wear Properties

**Figure 6:**
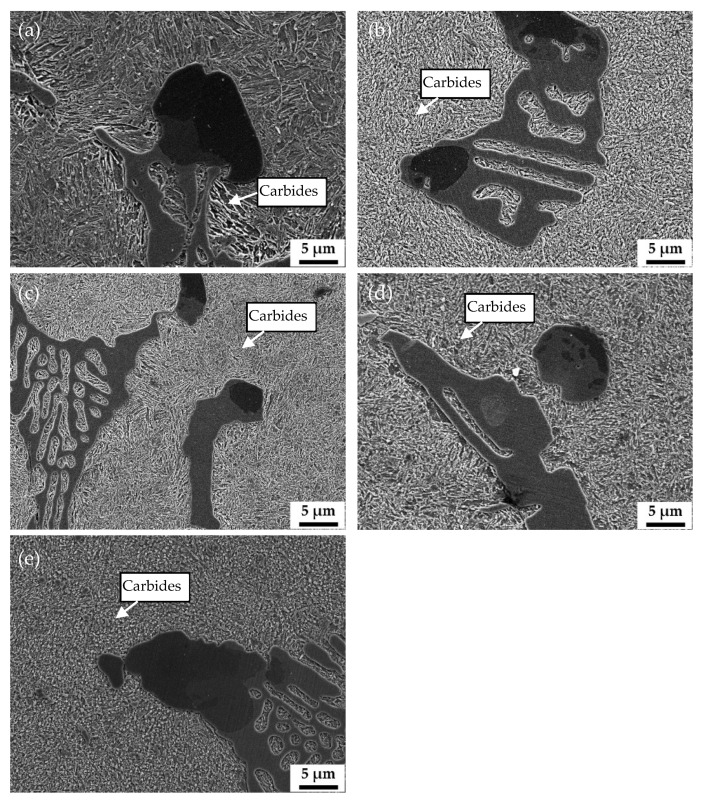
SEM images of V3 tempered at different temperatures: (**a**) 150 °C, (**b**) 250 °C, (**c**) 350 °C, (**d**) 450 °C, (**e**) 550 °C.

Figure 6 shows the effect of tempering temperature on microstructure and wear resistance of V3 after being quenched at 900 °C. It can be seen from Figure 6a that the matrix is a typical lath martensite. As the tempering temperature increases, the amount of carbide precipitation in the matrix increases, and the size of the carbide also increases, as shown in Figure 6b–e.

**Figure 7:**
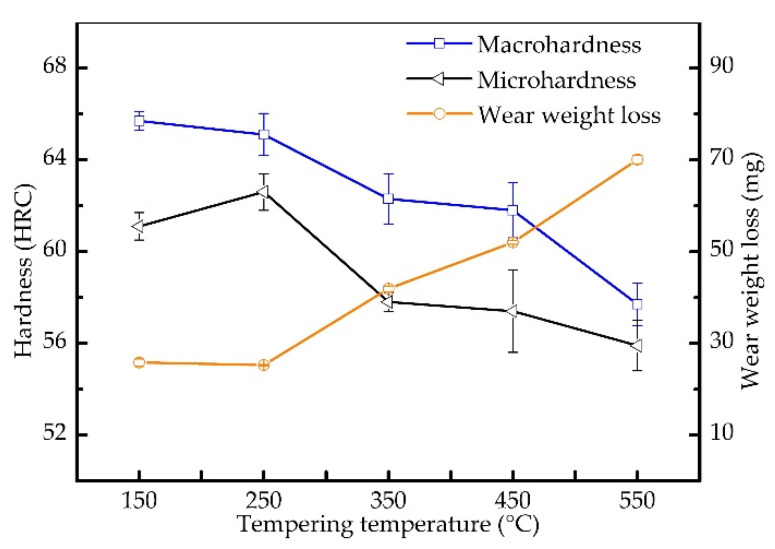
Macrohardness, microhardness of the matrix, and wear resistance of V3 tempered at different temperatures.

Figure 7 shows the macrohardness, microhardness of the matrix, and wear resistance of V3 tempered at different temperatures. As the tempering temperature increases, the macrohardness of the sample decreases. When the tempering temperature increases from 250 °C to 350 °C and 450 °C to 550 °C, the macrohardness decreases significantly. It can be seen from Figure 7 that as the tempering temperature increases, the microhardness of the matrix gradually decreases, except the microhardness of the specimen tempered at 250 °C. Solid solution strengthening, forest dislocation strengthening, and precipitation strengthening are the main mechanisms that affect the hardness of alloys during the tempering process. It can be inferred that the contribution of the three strengthening mechanisms to the hardness reaches a maximum when the sample is tempered at 250 °C, which benefits the wear resistance of the alloy. When the tempering temperature is less than 250 °C, the wear resistance of V3 is excellent, and the tempering temperature has no obvious effect on the wear resistance. When the tempering temperature exceeds 250 °C, the wear resistance decreases with the increase of the tempering temperature.

## 4. Discussion

The vanadium content of the low-vanadium wear-resistant alloys has a significant effect on the primary and eutectic phases, as shown in Figure 1. The primary phase is VC, and the eutectic phase is M_7_C_3_. The increase of vanadium content increases the number density of blocky VC, which is evenly distributed in the matrix. At the same time, due to the consumption of carbon by VC, the volume fraction and size of the eutectic phase (M_7_C_3_) decreases. It can be found that eutectic carbides usually nucleate at the primary phase. And this makes the eutectic phase in V3 small in size and evenly distributed. As we know, the primary and eutectic phases are completed at solidus temperature, i.e., around 1150 °C at the highest. Thus, they remain almost unchanged during further cooling or heat treatment. Therefore, the heat treatment of low-vanadium wear-resistant alloys is to adjust the microstructure of the matrix and improve the global performance.

Because of high carbon content, raising the quenching temperature increases not only the hardness of martensite, but also the austenite/martensite ratio. Hence the macro-hardness of the three alloys decreases as the quenching temperature increases, as shown in Figure 2. A large amount of high-carbon martensite will lead to a higher quenching stress, making the material brittle, whereas increasing the volume fraction of austenite will reduce the hardness and wear resistance of the alloy. Therefore, the choice of quenching temperature is important. After overall consideration, 900 °C was set as the quenching temperature for the low-vanadium wear-resistant alloys. Nevertheless, many quenching microcracks appeared in the microstructure of V1 and V2 after quenching.

**Figure 8:**
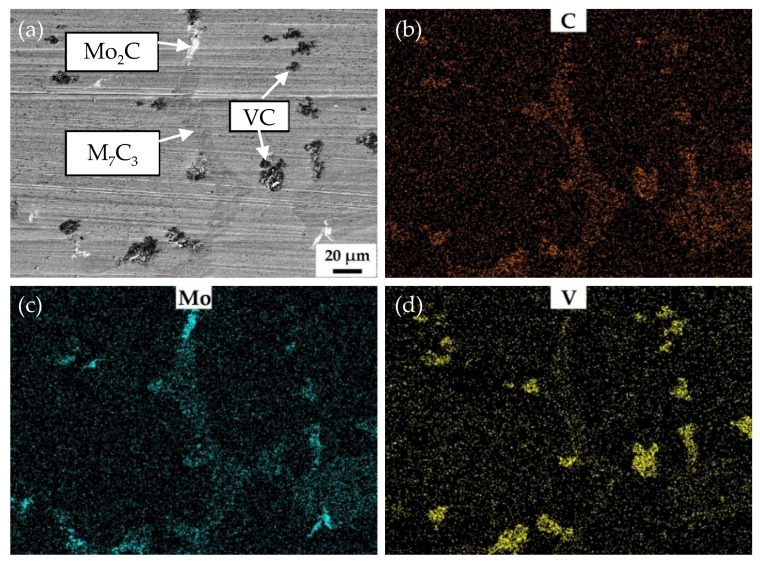
SEM and EDS mapping images of the surface of V3 quenched at 900 °C and tempered at 250 °C after abrasive wear test. (**a**) SEM image, (**b**), (**c**) and (**d**) EDS mapping images of C, Mo and V, respectively.

**Figure 9:**
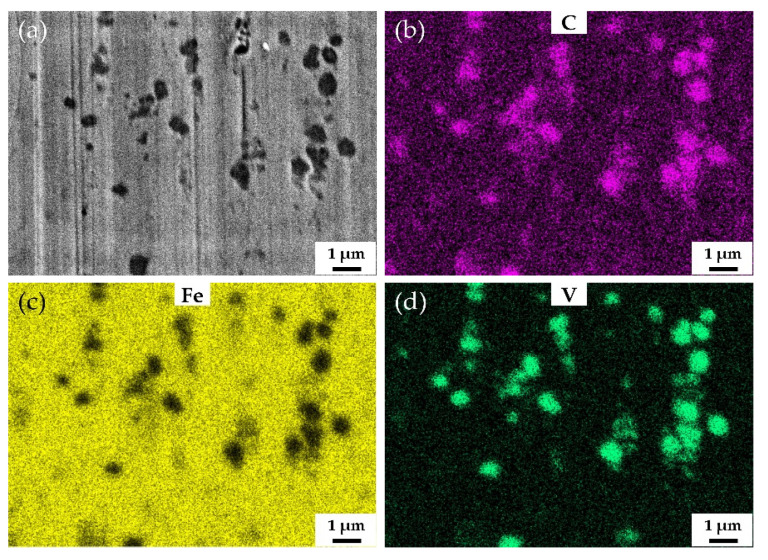
SEM and EDS mapping images of the surface of V3 quenched at 900 °C and tempered at 250 °C at high magnification after abrasive wear test. (**a**) SEM image, (**b**), (**c**) and (**d**) EDS mapping images of C, Fe and V, respectively.

**Figure 10:**
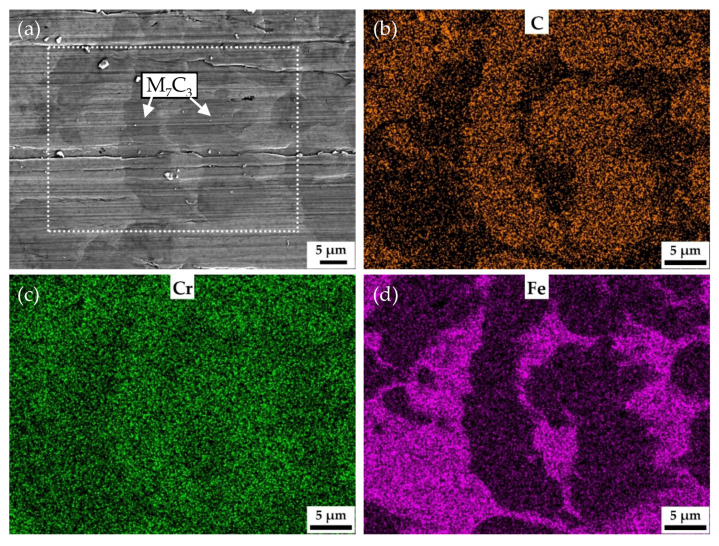
SEM and EDS mapping images of the surface of HCCI after abrasive wear test. The white dotted box is the area of EDS mapping. (**a**) SEM image, (**b**), (**c**) and (**d**) EDS mapping images of C, Cr and Fe, respectively.

The increase in vanadium content only brings about slight increase in the macro-hardness of the samples quenched at 900 °C and tempered at 250 °C. However, the vanadium content has significant effect on the wear resistance. The wear weight loss of V3 is only half the wear weight loss of HCCI. Figure 8 shows the SEM and EDS mapping images of the surface of V3 quenched at 900 °C and tempered at 250 °C after the abrasive wear test. As can be seen from Figure 8a, there are no scratches left on VC and Mo_2_C, which means that they are effective to impede wear induced by grooving or indenting because of their high hardness. Figure 9 shows the behavior of submicron carbides in the matrix of V3 after abrasive wear. It can be seen that the grooving of the abrasive was terminated by submicron VC in the matrix, which means these submicron VC can effectively hinder the abrasive cutting of the matrix. Figure 10 shows the SEM and EDS mapping images of the surface of HCCI after the abrasive wear test. It can be found that there is no obvious difference between the scratches on the matrix and the carbides (M_7_C_3_). This indicates that the blocky VC and submicron VC evenly distributed in the matrix is the key to improving its abrasive wear performance.

The tempering temperature affects the hardness and wear resistance of the low-vanadium wear-resistant alloy by changing the matrix microstructure. In general, the performance of wear-resistant materials is mainly determined by two constituents, i.e., hard phase and metal matrix. The two constituents of wear resistant materials serve different purposes: the hard phases are to impede wear induced by grooving or indenting mineral particles. In addition to resisting wear, the metal matrix is meant to provide adequate strength and toughness for the material. Both properties depend on the amount, size, and distribution of hard phases as well as on the hardness and fracture toughness of both constituents and the bond between them. As the tempering temperature increases, the number density and size of carbides precipitated in martensite increase. The precipitation of the tempered carbides reduces the hardness of the matrix and does not significantly promote the improvement of wear resistance, which leads to the reduction of the global wear resistance of the material. Therefore, tempering is only used to improve the toughness of the matrix for low-vanadium wear-resistant alloys.

## 5. Conclusions

To weigh different wear-resistant materials one has to consider their expected performance in service as well as the feasibility of manufacturing and the cost-effectiveness. For the sake of obtaining a more reasonable wear-resistant material, a class of low-vanadium wear-resistant alloys was designed and characterized. In this study, following conclusions can be drawn:

1. As the vanadium content increases, the amount of primary carbides (VC) increases, and the amount of eutectic carbides (M_7_C_3_) decreases, resulting in a decrease in the size of M_7_C_3_ and a more homogeneous distribution of M_7_C_3_.
2. Because of the high carbon content in the matrix of low-vanadium wear-resistant alloys, increasing the quenching temperature will lead to the increase of the amount of austenite. This will reduce the macro-hardness of the alloys, which may not be conducive to the wear property.
3. Tempering treatment is used to improve the toughness of the matrix, but when the tempering temperature exceeds 250 °C, a large amount of carbides will precipitate in the matrix, which makes the hardness and wear resistance of the material decrease significantly.
4. As the vanadium content increases, the wear properties of low-vanadium wear-resistant alloys are improved. V3 quenched at 900 °C and tempered at 250 °C, possessing VC, Mo_2_C, and M_7_C_3_ distributed in the martensite matrix, displayed a wear resistance two times better than the HCCI.
5. The VC in the low-vanadium wear-resistant alloys plays a vital role in improving the wear resistance. The size of VC in V3 has a bimodal distribution. The size of blocky VC, usually attached to M_7_C_3_, is about 10 μm. The size of VC distributed in the martensite matrix is submicron. Both forms of VC are effective to impede wear induced by grooving or indenting because of their high hardness.
6. Increasing the vanadium content can not only increase the volume fraction of VC, which possess excellent wear resistance performance, but also refine the M_7_C_3_. Thus, increasing the vanadium content coupling with appropriate heat treatment can help for developing new alloys with outstanding wear resistance.

## Figures and Tables

**Table 1:** Chemical compositions of low-vanadium wear-resistant alloys and HCCI (weight percent).

Element	C	Cr	Mo	V	Si	Mn	P	S	Fe
V1	2.15	3.82	2.62	0.98	0.71	0.80	0.037	0.026	Bal.
V2	2.17	3.85	2.65	1.86	0.68	0.75	0.014	0.020	Bal.
V3	2.15	3.78	2.57	3.00	0.72	0.77	0.037	0.028	Bal.
HCCI	3.06	23.01	0.52	-	0.62	1.15	0.062	0.053	Bal.

## Data Availability

The data presented in this study are available on request from the corresponding author.
